# Endocrine-Disrupting Chemicals (EDCs): *In Vitro* Mechanism of Estrogenic Activation and Differential Effects on ER Target Genes

**DOI:** 10.1289/ehp.1205951

**Published:** 2013-02-05

**Authors:** Yin Li, Colin J. Luh, Katherine A. Burns, Yukitomo Arao, Zhongliang Jiang, Christina T. Teng, Raymond R. Tice, Kenneth S. Korach

**Affiliations:** 1 Receptor Biology Section, Laboratory of Reproductive and Developmental Toxicology, Division of Intramural Research, National Institute of Environmental Health Sciences (NIEHS), National Institutes of Health (NIH), Department of Health and Human Services (DHHS), Research Triangle Park, North Carolina, USA; 2 Biomolecular Screening Branch, Division of National Toxicology Program, NIEHS, NIH, DHHS, Research Triangle Park, North Carolina, USA

**Keywords:** E_2_, EDCs, ERα, ERβ, ERE, ER target genes. *Environ Health Perspect* 121:459–466 (2013)

## Abstract

Background: Endocrine-disrupting chemicals (EDCs) influence the activity of estrogen receptors (ERs) and alter the function of the endocrine system. However, the diversity of EDC effects and mechanisms of action are poorly understood.

Objectives: We examined the agonistic activity of EDCs through ERα and ERβ. We also investigated the effects of EDCs on ER-mediated target genes.

Methods: HepG2 and HeLa cells were used to determine the agonistic activity of EDCs on ERα and ERβ via the luciferase reporter assay. Ishikawa cells stably expressing ERα were used to determine changes in endogenous ER target gene expression by EDCs.

Results: Twelve EDCs were categorized into three groups on the basis of product class and similarity of chemical structure. As shown by luciferase reporter analysis, the EDCs act as ER agonists in a cell type– and promoter-specific manner. Bisphenol A, bisphenol AF, and 2-2-bis(*p*-hydroxyphenyl)-1,1,1-trichloroethane (group 1) strongly activated ERα estrogen responsive element (ERE)-mediated responses. Daidzein, genistein, kaempferol, and coumestrol (group 2) activated both ERα and ERβ ERE-mediated activities. Endosulfan and kepone (group 3) weakly activated ERα. Only a few EDCs significantly activated the “tethered” mechanism via ERα or ERβ. Results of real-time polymerase chain reaction indicated that bisphenol A and bisphenol AF consistently activated endogenous ER target genes, but the activities of other EDCs on changes of ER target gene expression were compound specific.

Conclusion: Although EDCs with similar chemical structures (in the same group) tended to have comparable ERα and ERβ ERE-mediated activities, similar chemical structure did not correlate with previously reported ligand binding affinities of the EDCs. Using ERα-stable cells, we observed that EDCs differentially induced activity of endogenous ER target genes.

Many natural and synthetic chemicals have been reported to disrupt the normal function of the endocrine system (Henley and Korach 2010). These compounds, classified as endocrine-disrupting chemicals (EDCs), interfere with hormone biosynthesis, metabolism, or action, which can result in deviation from normal homeostatic control and can alter normal development and reproduction (Diamanti-Kandarakis et al. 2009). Many known EDCs influence the activity of the estrogen receptors (ERs) and alter their function in *in vitro* and *in vivo* model systems (Diamanti-Kandarakis et al. 2009). Estrogens play an essential role in the growth, differentiation, and homeostasis of a number of target tissues, including reproductive tracts (both male and female), mammary glands, bone, brain, and liver (Katzenellenbogen 1996; Katzenellenbogen et al. 1997; Lubahn et al. 1993; McDonnell and Norris 2002; Nilsson et al. 2001; Pettersson and Gustafsson 2001). The biological effects of estrogen (E_2_) are mediated through two ERs, ERα and ERβ, which belong to the nuclear receptor superfamily of ligand-inducible transcription factors (Hall and McDonnell 2005). There are two major mechanisms of ER-mediated transcriptional gene regulations. In the classical mechanism, ERs directly bind to estrogen responsive elements (EREs) located in the promoter region of target genes. The nonclassical mechanism is the “tethered” mechanism, which involves the ERs regulating gene expression by associating with other transcription factors such as c-Jun and c-Fos, which bind the DNA but not with direct ER–DNA binding (Björnström and Sjöberg 2005; Hall and McDonnell 2005; O’Lone et al. 2004).

Estrogens regulate a large number of target genes through the ER. *PR* (progesterone receptor) and *pS2* are the well-known ER target genes (Berry et al. 1989; Katzenellenbogen 2000). *GREB1* (gene regulation by estrogen in breast cancer 1) and *SPUVE* (a member of the trypsin family of serine proteases) have been reported to be ER-responsive genes (Henley et al. 2009; Reid et al. 2005). Recently, we discovered that these target genes are induced by bisphenol A (BPA) and bisphenol AF (BPAF), a fluorinated derivative of BPA, and that the gene expression changes are compound specific (Li et al. 2012). *WISP2* (WNT1-inducible-signaling pathway protein 2) gene expression is enhanced by important modulators of human breast cancer cell proliferation such as E_2_, progesterone, and epidermal growth factor. These effects, inhibited by appropriate antagonists, indicate that steroids and growth factor–induced up-regulation of *WISP-2* may be mediated through ERs (Dhar et al. 2007). *SDF-1* (stromal cell-derived factor 1) was identified as a key target of estrogens in ER-positive breast and ovarian cells (Hall and Korach 2012). The correlation between chemical structure and the functionality of the EDCs through the ERs, as well as the effects of EDCs on ER target genes, remains unclear.

BPA, BPAF, and other EDCs with a similar chemical structure have been frequently studied. BPA is widely used in the manufacturing of polycarbonate plastics and as a nonpolymer additive to other plastics (Wetherill et al. 2007). BPA uptake in humans from food, beverages, and the environment has been measured in adult and fetal serum at a range of 0.5–40 nM (Welshons et al. 2006). BPAF is used in polycarbonate copolymers in high-temperature composites, electronic materials, and specialty polymer applications (Akahori et al. 2008; Perez et al. 1998). 2,2-bis(*p*-Hydroxyphenyl)-1,1,1-trichloroethane (HPTE), an estrogenic metabolite of the pesticide methoxychlor, has estrogenic effects similar to that of BPA (Borgeest et al. 2002; Hewitt and Korach 2011; Klotz et al. 2000). 4-*n*-Nonylphenol (4n-NP) is a resistant alkylphenol that is degraded from alkylphenol ethoxylates and is generally present in food (Guenther et al. 2002; Ying et al. 2002).

Several natural products (known as phytoestrogens) have been identified as estrogenic EDCs. Daidzein (Dai) is a soy-derived isoflavone that originates from plants and herbs (Dang 2009). Genistein (Gen), another isoflavone) is found in a number of plants, including lupin, fava beans, soybeans, kudzu, and psoralea (Dang 2009). Kaempferol (Kaem) is a flavonoid/isoflavone isolated from tea, broccoli, grapefruit, apples, and other plant sources (Calderón-Montaño et al. 2011). Apigenin (Api) is a flavonoid/flavone used to dye wool (Ferreira et al. 2006). Coumestrol (Coum), an organic compound in the class of phytochemicals known as coumestans, has classically been categorized as a phytoestrogen because it binds to the ER (Markaverich et al. 1995).

Other estrogenic EDCs of interest that have a common structural component include endosulfan (Endo), kepone (Kep), and 1-bromopropane (1-BP). Endo is a fluorinated organic insecticide, and animal studies have indicated that it affects the male reproductive system (Murray et al. 2001). Kep, also known as chlordecone, is a chlorinated polycyclic hydrocarbon insecticide and fungicide. *In vitro* studies have shown that Kep has ligand binding affinity to ERα (van Lipzig et al. 2004). 1-BP, categorized as a high production volume chemical, is used in the manufacture of pharmaceuticals, pesticides, and other chemicals (Anderson et al. 2010).

In the present study, we used two ER-negative cell lines, HepG2 and HeLa, to analyze the effects of 12 estrogenic EDCs—which were grouped based on chemical structure and product class—on the estrogenic ERE- and AP-1/Sp1–mediated responses of ERα and ERβ. Using Ishikawa cells that stably express ERα, we evaluated changes in endogenous ER target gene expression after EDC treatment.

## Materials and Methods

*Chemicals.* 17β-Estradiol (E_2_) was purchased from Sigma-Aldrich (St. Louis, MO), and ICI 182,780 (ICI) was obtained from Tocris Bioscience (Ellisville, MO). The 12 EDCs used in this study were provided by the Midwest Research Institute (Kansas City, MO) via a contract with the National Toxicology Program. The chemical names, Chemical Abstracts Services Registry Numbers, and the sources are summarized in Supplemental Material, Table S1 (http://dx.doi.org/10.1289/ehp.1205951).

**Figure 1:**
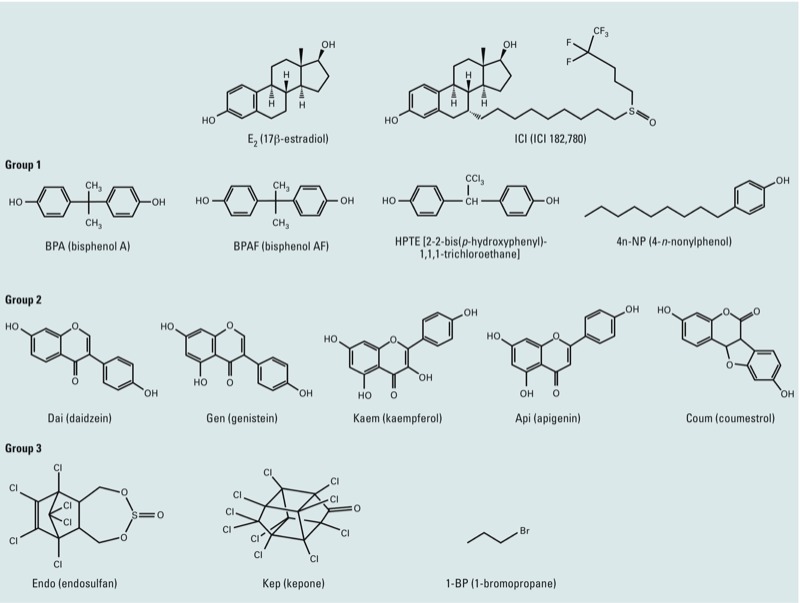
The chemical structures of EDCs tested in this study.

*EDC groups.* The 12 EDCs were categorized into three groups based on their chemical and product classes (Figure 1, Table 1). Group 1 consists of BPA, BPAF, HPTE, and 4n-NP because of their shared bisphenol or phenol group. Dai, Gen, Kaem, Api, and Coum, all from natural products, comprise group 2; they each contain flavonoid, isoflavone, or phenol. Group 3 includes Endo, Kep, and 1-BP because they each contain organochlorine or organobromine in their chemical structures. Group 3 EDCs have traditionally been used as pesticides or chemical intermediates.

**Table 1:** EDCs used in this study.

EDC	Chemical class	Product class	MW
E2 (17β-estradiol)	Phenolic steroid estrene	Hormone	272.38
ICI (ICI 182,780)	Phenolic steroid	Pharmaceutical	606.77
Group 1
BPA (bisphenol A)	Diphenylalkane, bisphenol, phenol	Chemical intermediate	228.29
BPAF (bisphenol AF)	Diphenylalkane, bisphenol, phenol	Chemical intermediate	336.23
HPTE [2-2-bis(p-hydroxyphenyl)-1,1,1-trichloroethane]	Diphenylalkane, bisphenol, phenol	Chemical intermediate	317.59
4-n-NP (4-n-nonylphenol)	Alkylphenol, phenol	Chemical intermediate	220.35
Group 2
Dai (daidzein)	Flavanoid, isoflavone, phenol	Natural product	254.23
GEN (genistein)	Flavanoid, isoflavone, phenol	Natural product	270.24
Kaem (kaempferol)	Flavanoid, isoflavone, phenol	Natural product	286.23
Api (apigenin)	Flavanoid, flavone, phenol	Natural product	270.24
Coum (coumestrol)	Flavanoid, isoflavone, phenol	Natural product	282.22
Group 3
Endo (endosulfan)	Organochlorine	Pesticide	406.93
Kep (kepone)	Organochlorine	Pesticide	490.64
1-BP (1-bromopropane)	Organochlorine	Chemical intermediate	122.99
MW, molecular weight.			

*Plasmids.* pcDNA vector plasmid was purchased from Promega (Madison, WI), pRL-TK vector plasmid from Invitrogen (Carlsbad, CA), and 7×AP-1 Luc from Stratagene (La Jolla, CA). pcDNA/mouse wild-type (WT)-ERα (pcDNA/ERα) and pcDNA/ΔNmERβ310G (former pcDNA/mouse WT-ERβ) have been described previously (Mueller et al. 2003). The full-length mouse ERβ expression plasmid, pcDNA/WT-ERβ, was generated as described in Supplemental Material, p. 3 (http://dx.doi.org/10.1289/ehp.1205951). The luciferase reporters 3×ERE (modified reporter) and pS2ERE (endogenous pS2 gene reporter) have been described previously (Hall et al. 2002). The following reporters were gifts: pRSV/c-Jun (M. Karin, University of California, San Diego, La Jolla, CA), -73Col AP-1 Luc (D.P. McDonnell, Duke University, Durham, NC) and p21Sp1 Luc (J.L. Jameson, University of Pennsylvania, Philadelphia, PA).

*Cell lines and tissue culture.* The HepG2 human hepatocellular cancer cell line and the HeLa cervical epithelial cancer cell line (both ER negative) were purchased from ATCC (Manassas, VA). The human endometrial adenocarcinoma stable cell lines Ishikawa/vector (Ishikawa/vec) and Ishikawa/WT ERα (Ishikawa/ERα) have been described previously (Burns et al. 2011; Li et al. 2012). HepG2 cells were maintained in phenol red–free minimum essential medium (MEM; Invitrogen) supplemented with 10% fetal bovine serum (FBS; Gemini Bio Products, West Sacramento, CA) and 4 mM l-glutamine (Invitrogen). HeLa cells were maintained in phenol red–free Dulbecco’s modified Eagle medium (DMEM; Invitrogen) supplemented with 10% FBS and 4 mM l-glutamine. The stable cell lines Ishikawa/vec and Ishikawa/ERα were maintained in phenol red–free DMEM:F12 medium (Invitrogen) supplemented with 10% FBS and geneticin (G418; 1 mg/mL; Invitrogen). For serum-starved conditions, 10% HyClone charcoal/dextran-stripped FBS (Thermo Scientific, Waltham, MA) was substituted for FBS in the medium (starve medium).

*Transient transfection and luciferase assay.* HepG2 and HeLa cells were seeded in 24-well plates with starve medium overnight. A total of 0.5 μg of DNA, including 0.2 μg of expression plasmid, 0.2 μg of reporter plasmid, and 0.1 μg of pRL-TK plasmid, were transfected overnight using Effectene transfection reagent (QIAGEN, Valencia, CA) according to the manufacturer’s protocol. E_2_, ICI, and EDCs were dissolved in 100% ethanol (EtOH) before being diluted in media. The final EtOH concentration was 0.01%. The cells were changed to fresh starve medium; after 8 hr, cells were treated with EtOH vehicle (control), 10 nM E_2_, 100 nM ICI, or EDCs for 18 hr. For experiments with pRSV/c-Jun on 7×AP-1 Luc, cells were transfected with a total of 0.7 μg of DNA, including 0.2 μg ERα or ERβ, 0.2 μg pRSV/c-Jun, 0.2 μg 7×AP-1 Luc, and 0.1 μg pRL-TK plasmids. Luciferase assays were performed using the Dual Luciferase Reporter Activity System (Promega, Madison, WI). Transfection efficiency was normalized by renilla luciferase using pRL-TK plasmid. All experiments were repeated at least three times. Data represent mean fold change (± SE; *n* = 3) relative to the control.

*RNA extraction and real-time polymerase chain reaction (PCR).* Ishikawa/vec and Ishikawa/ERα cells were cultured in starve medium for 2 days and then treated with 10 nM E_2_, 100 nM EDCs, or EtOH vehicle (control) for 18 hr. Total RNA was extracted using the RNeasy Mini Kit (QIAGEN). First-strand cDNA synthesis was performed using Superscript reverse transcriptase (Invitrogen) according to the manufacturer’s protocol. The mRNA levels of ER target genes were measured using SYBR green assays (Applied Biosystems, Carlsbad, CA). The Genbank accession numbers (http://www.ncbi.nlm.nih.gov/genbank/) and sequences of primers used for real-time PCR were as follows: human *PR* (NM_000926.4): forward 5´-GACG​TGGA​GGGC​GCAT​AT-3´, reverse 5´-GCAG​TCCG​CTGT​CCTT​TTCT-3´; human *pS2/TFF1* (NM_003225.2): forward 5´-GCCC​TCCC​AGTC​TGCA​AATA-3´, reverse 5´-CTGG​AGGG​ACGT​CGAT​GGTA-3´; human *GREB1* (NM_014668): forward 5´-CAAA​GAAT​AACC​TGTT​GGCC​C-3´, reverse 5´-GACA​TGCC​TGCG​CTCT​CATA​C-3´; human *SPUVE* (NM_007173): forward 5´-ATGC​CCGA​GCAG​ATGA​AATT-3´, reverse 5´-CCAA​CCCT​TGGG​CACA​TG-3´; human *WISP2* (NM_003881): forward 5´-TGAG​CGGC​ACAC​CGAA​GAC-3´, reverse 5´ACAG​CCAT​CCAG​CACC​AG-3´; human *SDF-1* (NM_000609): forward 5´-GTGG​TCGT​GCTG​GTCC​TC-3´, reverse 5´-GATG​CTTG​ACGT​TGGC​TCTG​-3´. Cycle threshold (Ct) values were obtained using the ABI PRISM 7900 Sequence Detection System and analysis software (Applied Biosystems, Foster City, CA). Each sample was normalized to its β-actin transcript content: forward 5´-GACA​GGAT​GCAG​AAGG​AGAT​CAC-3´, reverse 5´-GCTT​CATA​CTCC​AGCA​GG-3´. The experiments were repeated three times, and results are presented as the mean fold change (± SE; *n* = 3) relative to control (vehicle-treated) Ishikawa/vec cells.

*Statistical analysis*. One-way analysis of variance (ANOVA) with Dunnett’s multiple comparison test and two-way ANOVA with Bonferroni posttests were performed using GraphPad Prism, version 6.00 (GraphPad Software Inc., La Jolla, CA).

## Results

*ERE-mediated estrogenic activation of ER*α *and ER*β *by EDCs.* To evaluate the ERE-mediated transcriptional activity of ERα and ERβ, we examined promoter activation in two ER-negative cell lines, HepG2 and HeLa. The two luciferase reporters, 3×ERE (modified reporter) and pS2ERE (endogenous pS2 gene reporter) (Hall et al. 2002) were used to determine the differential effects of these EDCs. First, we confirmed that there was no reporter activation in either of these ER-negative cell lines after stimulation with 10 nM E_2_ (data not shown). Because we observed estrogenic effects of BPA and BPAF at 100 nM concentrations in cells with WT-ERα (Li et al. 2012), we used this concentration to examine all of the EDCs.

**Figure 2:**
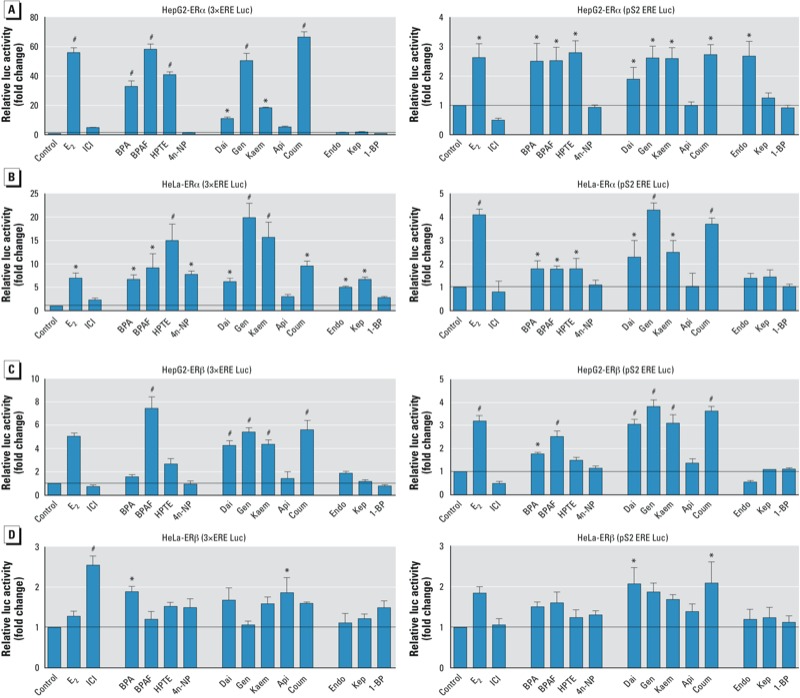
EDCs act as agonists on ERα (*A,B*) and ERβ (*C,D*) to activate the classical mechanism (ERE) in HepG2 and HeLa cells. (*A,B*) Activation of ERα in HepG2 (*A*) and HeLa (*B*) cells transfected with ERE-luc (3×ERE or pS2 ERE), pRL-TK, and pcDNA/WT-ERα or pcDNA/WT-ERβ plasmids overnight, and then treated with vehicle (control), 10 nM E_2_, 100 nM ICI, or EDCs for 18 hr; ERα ERE-mediated activation was detected by luciferase reporter assays. (*C,D*) Activation of ERβ in HepG2 (*C*) and HeLa (*B*) cells transfected with ERE-luc (3×ERE or pS2 ERE), pRL-TK, and pcDNA/WT-ERβ plasmids overnight and then treated with vehicle (control), 10 nM E_2_, 100 nM ICI, or EDCs for 18 hr; ERβ ERE-mediated activation was detected by luciferase reporter assays. See “Materials and Methods” for details of the experiments. Data shown represent mean fold change (± SE) relative to the control. *p < 0.05, **p < 0.01, and ^#^*p* < 0.001, compared with control.

The ERα ERE-mediated activation by EDCs is shown in Figure 2A and B. HepG2 cells were highly responsive to E_2_, with up to 50-fold increases in 3×ERE-mediated transactivation (Figure 2A, left). Group 1 and group 2 EDCs strongly activated ERα 3×ERE-mediated responses in HepG2 cells, with the exception of 4n-NP and Api at 100 nM. However, no activation was seen with group 3 EDCs at 100 nM concentration. Even though the pS2ERE reporter had weaker response to E_2_, similar responses were obtained with EDC treatments (Figure 2A, right). Interestingly, induction with Endo in HepG2 cells was detected only with the pS2ERE reporter. In HeLa cells, all EDCs, with the exception of Api and 1-BP, significantly induced 3×ERE-mediated activity (Figure 2B, left). However, only three EDCs from group 1 (BPA, BPAF, and HPTE) and four EDCs from group 2 (Dai, Gen, Kaem, and Coum) induced pS2ERE-mediated activation (Figure 2B, right).

For ERβ ERE-mediated activation, both ERE reporters exhibited responses to E_2_ in HepG2 cells (Figure 2C). BPAF (group 1) and Dai, Gen, Kaem, and Coum (group 3) have strong activation of ERβ 3×ERE and pS2ERE-mediated responses in HepG2 cells. In HeLa cells, ICI, BPA, and Api induced activity with the 3×ERE reporter, and Dai and Coum induced activity with the pS2ERE reporter (Figure 2D). However, group 3 EDCs did not activate ERβ ERE-mediated activity in HepG2 or HeLa cells. To confirm that the reporter activation of EDCs through ERα and ERβ was ER specific, we used ICI, a pure ER antagonist, to block activity (data not shown). These results demonstrate that EDCs can activate ERE-mediated transcription in different cell types via ERα and ERβ in cell-type and promoter-selective manners, and that the structural similarities among the EDCs correlate to their estrogenic activity.

*The effects of EDCs on AP-1 and Sp1 reporters for ER*α *and ER*β. To verify the effects of the EDCs on the “tethered” mechanism of ERα and ERβ, we used the 7×AP-1 reporter (Jakacka et al. 2001; Kushner et al. 2000; Webb et al. 1995), the -73Col AP-1 reporter (Sharma and Richards 2000), and the p21Sp1 reporter (De Siervi et al. 2004) to test AP-1/Sp1–mediated activation.

**Figure 3:**
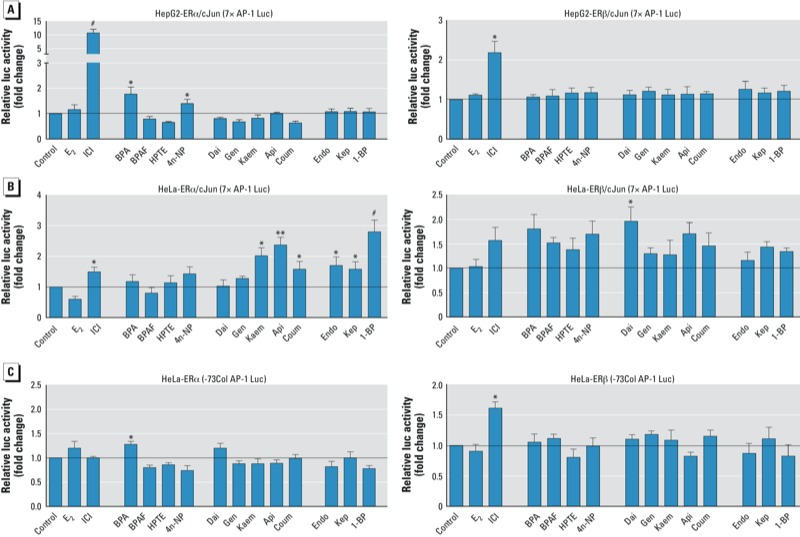
EDCs act as agonists on ERα and ERβ to activate the tethered mechanism (AP-1). (*A*) Effects of EDCs on ERα 7×AP-1 Luc reporter activity in HepG2 (top) and HeLa (bottom) cells transfected with 7×AP-1 Luc, pRL-TK, vehicle (control), 10 nM E_2_, 100 nM ICI, or EDCs for 18 hr; ER AP-1–mediated activation was detected by luciferase reporter assays. (*B*) Effects of EDCs on ERα‑ and ERβ-73Col AP-1 Luc reporter activity in HeLa cells transfected with -73Col AP-1 Luc, pRL-TK, and pcDNA/WT-ERα or -ERβ plasmids overnight and then treated with vehicle (control), 10 nM E_2_, 100 nM ICI, or EDCs for 18 hr; ER -73Col AP-1–mediated activation was detected by luciferase reporter assays. See “Materials and Methods” for details of the experiments. Data shown represent mean fold change (± SE) relative to the control. *p < 0.05, **p < 0.01, and ^#^*p* < 0.001, compared with control.

To detect the ligand-dependent/AP-1–mediated reporter activity, the 7×AP-1 Luc reporter, c-Jun, and ERα or ERβ were co-transfected into the cells. For ERα activation, ICI, as a positive control (Kushner et al. 2000), had a stronger response in HepG2 cells than in HeLa cells (Figure 3A,B). ICI induced the 7×AP-1 reporter activity > 10-fold in HepG2 cells; however, only BPA and 4n-NP showed weak activity (Figure 3A, left). In HeLa cells, Kaem, Api, and Coum (group 2) and all group 3 EDCs activated the 7×AP-1 reporter (Figure 3B, left). For ERβ, only ICI induced 7×AP-1 reporter activity in HepG2 cells (Figure 3A, right). All EDCs induced minor ERβ/7×AP-1 reporter activity in HeLa cells, but only Dai showed significant activation (Figure 3B, right).

Using the -73Col AP-1 reporter in HeLa cells, only BPA (group 1) showed weak activity via ERα (Figure 3C, left) and ICI induced weak activity via ERβ (Figure 3C, right). However, we observed no activation for either ER in EDC-treated HepG2 cells (data not shown). Last, for the p21Sp1 reporter using either ERα or ERβ, the induction levels were insignificant to discriminate agonistic tendencies in either HepG2 and HeLa cells (data not shown). These findings suggest that EDCs induce weak activity for the “tethered” mechanism in a cell-type and promoter-specific manner.

**Figure 4:**
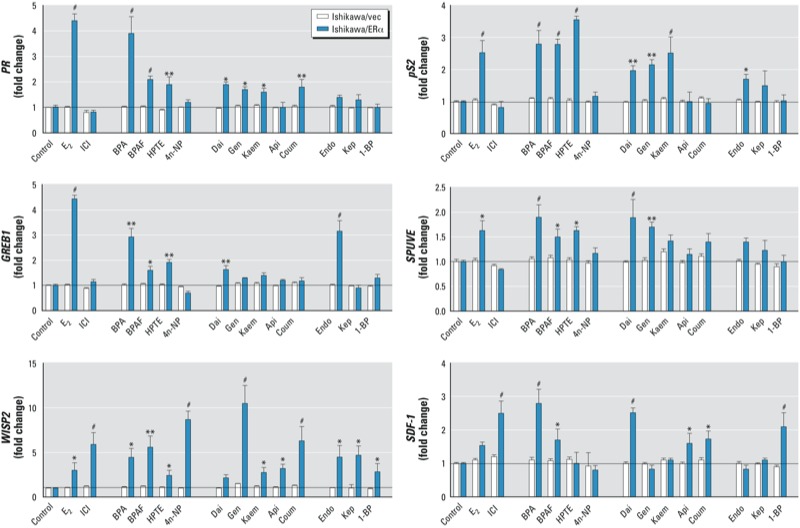
Effects of EDCs on expression of the ER target genes of *PR*, *pS2*, *GREB1, SPUVE*, *WISP2*, and *SDF-1* in Ishikawa/vector and Ishikawa/ERα cells. Total RNA was extracted from Ishikawa/vec or Ishikawa/ERα cells after treatment with vehicle (control), 10 nM E_2_, 100 nM ICI, or EDCs for 18 hr; mRNA levels of *PR*, *pS2*, *GREB1*, *SPUVE*, *WISP2*, and *SDF-1* were quantified by real-time PCR. Data were normalized to β‑actin and represent mean fold change (± SE) relative to control Ishikawa/vec cells. *p < 0.05, **p < 0.01, and ^#^*p* < 0.001, compared with control Ishikawa/vec cells.

*The effects of EDCs on expression of ER target genes.* To characterize the ER-dependent response of EDCs, we examined their effects on ERα target genes (*PR*, *pS2*, *GREB1*, *SPUVE*, *WISP2*, and *SDF-1*) using real-time PCR in Ishikawa/ERα stable cells (Burns et al. 2011; Li et al. 2012). Data were normalized to β-actin and are presented in Figure 4 as fold change in gene expression, relative to the vehicle control. The group 1 EDCs BPA and BPAF significantly induced the endogenous ERα target genes *PR*, *pS2*, *GREB1*, *SPUVE*, *WISP2*, and *SDF-1*, and HPTE significantly induced all of these genes except *SDF-1*. 4n-NP significantly induced only *WISP2*. The group 2 EDCs varied in their induction of ER target genes: Dai significantly induced *PR*, *pS2*, *GREB1*, *SPUVE*, and *SDF-1*; Gen significantly induced *PR*, *pS2*, *SPUVE*, and *WISP2*; Kaem significantly induced *PR*, *pS2,* and *WISP2*; Api significantly induced *WISP2* and *SDF-1*; and Coum significantly induced *PR*, *WISP2*, and *SDF-1*. Similarly, the group 3 EDCs varied in their induction of target genes: Endo significantly activated *pS2*, *GREB1*, and *WISP2*; Kep significantly activated only *WISP2*; and 1-BP significantly activated *WISP2* and *SDF-1*. In contrast, expression of target genes in the Ishikawa/vector stable cells did not change with any EDC treatments, demonstrating that the changes in target gene expression are ER dependent. These results indicate that EDCs affect many aspects of transcriptional regulation in this *in vitro* cell culture model; this information may be helpful in identifying compound-specific genes that are involved in cellular signaling responses.

## Discussion

Many EDCs adversely affect estrogen signaling by interacting with two ERs: ERα and ERβ. We are interested in defining the roles of ERs in mediating cellular and physiological responses to EDCs based on the similarities in chemical structure. One of the most significant findings of our study is that the structural similarities of the EDCs correlate with their estrogenic activity for ERs. The 3×ERE Luc reporter contains a series of three 13-base-pair inverted repeats (GGTCAnnnTGACC; perfect ERE), whereas pS2ERE Luc, derived from the human pS2 gene promoter, contains an imperfect ERE sequence (GGTCAnnnTGGCC) and several AP-1 sites (Hall et al. 2002). Using these two reporters, we found that BPA, BPAF, and HPTE (group 1 EDCs) strongly activated ERα ERE-mediated responses, but these compounds did not activate ERβ. BPA binds strongly to estrogen-related receptor γ (ERR-γ), an orphan receptor that behaves as a constitutive activator of transcription, but only weakly binds to the ERs (Matsushima et al. 2007). In contrast to our reporter assays, *in vitro* receptor-binding analysis shows that the ligand binding activity of BPAF and HPTE is three times stronger for ERβ than for ERα (Matsushima et al. 2010). The group 2 EDCs Dai, Gen, Kaem, and Coum activated both ERα and ERβ ERE-mediated activity. In fact, Dai, Gen, Kaem, and Coum were reported to be more competitive than E_2_ for binding to ERβ (Hwang et al. 2006; Kuiper et al. 1998). These results from *in vitro* expreriments indicate that the ERE-mediated activity of these EDCs does not correlate with their receptor ligand binding activity. In a recent analysis, we found that a mouse ERβ expression plasmid used previously (pcDNA/ΔNER_β_G) had a mutation of 310 glutamic acid (E) to glycine (G). Using this mutated ERβ plasmid, we found that BPAF (group 1 EDC) and Kaem (group 2 EDC) lost the majority of ERE-mediated activity in HepG2 cells relative to full-length ERβ [see Supplemental Material, Figure S1 (http://dx.doi.org/10.1289/ehp.1205951)]. Additionally, Endo and Kep (group 3) exhibited weak activation of ERα in a cell type–specific manner (only in HeLa cells), suggesting that cell type–specific factors are involved in regulating ER ERE-mediated activity.

EDCs activate the nonclassical “tethered” ER mechanism (AP-1/Sp1–mediated ER activation) in a manner not correlative to chemical structure similarity There is growing literature supporting E_2_’s ability to affect gene expression through the nonclassical “tethered” mechanism, which involves ER modulating the activity of other transcription factors such as AP-1 and Sp1. Webb et al. (1995) first reported the ER activation of the -73Col AP-1 promoter reporter construct, derived from the human collagenase promoter. Using three different reporters (7×AP-1, -73Col AP-1, and p21Sp1 Luc), we found that ERα AP-1–mediated activation in HeLa cells was variable; Kaem, Api, and Coum (group 2) and Endo, Kep, and 1-BP (group 3) showed activity with the 7×AP-1 reporter. In contrast, all EDCs induced minor activity for the ERβ “tethered”-mediated mechanism with the 7×AP-1 reporter in HeLa cells, but only Dai showed significant activation. Furthermore, we observed no activation of the -73Col AP-1 reporter via ERα or ERβ with EDC treatment, except for BPA in HeLa cells and ICI in HepG2 cells. Our data suggest that cell-specific coregulators may be involved in reporter activation by the EDCs in these cell lines. In addition, ER AP-1–mediated activation was observed in HepG2 cells only with ICI. Similar results were obtained with the mutated ERβ [see Supplemental Material, Figure S2 (http://dx.doi.org/10.1289/ehp.1205951)]. These data indicate that EDCs activate the nonclassical “tethered” ER mechanism in a manner not correlative to their chemical structure similarity and that ERβ AP-1–mediated activation of EDCs occurs only in a cell type- and promoter–specific manner.

EDCs induce ER target gene expression in a compound-specific manner. ERs, as transcription factors, are able to induce gene expression events sufficient for altered cellular responses, some of which include cell division and cancer progression. The advent of expression microarrays has allowed for the investigation of global gene expression changes after ligand treatment. Our laboratory has examined gene expression profiles of the estrogenic activity of BPA and HPTE in the mouse uterus, finding that similar target genes are induced by BPA, HPTE, and E_2_ 2 hr after treatment (Hewitt and Korach 2011). This demonstrates that there may be similar target genes in the uterus that are activated by EDCs and E_2_. The sequences of the DNA binding domains of ERα and ERβ are 97% similar, and ligand binding induces conformational changes to the ERs, promoting dimerization and high-affinity binding to EREs within the regulatory regions of target genes (Hall and McDonnell 2005); thus, we used Ishikawa cells stably expressing ERα (Burns et al. 2011; Li et al. 2012) to investigate several endogenous ER target genes, including *PR*, *pS2*, *GREB1*, *SPUVE*, *WISP2*, and *SDF-1*, after EDC treatments. We found that E_2_ induced expression of *PR*, *pS2*, and *GREB1* in this *in vitro* model. Our results showed that of the group 1 EDCs, BPA and BPAF significantly induced all six of the endogenous genes, and HPTE induced all of the genes except SDF-1. However, induction of target gene expression by group 2 and group 3 EDCs was target gene specific. More interestingly, ICI induced *WISP2* and *SDF-1*, suggesting that these two genes may have an AP-1–type regulating sequence. Future analysis of specific target gene promoters would be beneficial in understanding any similarities or differences in how the EDCs activate the ERs and elicit tissue-specific actions.

## Conclusions

In this study, we observed a correlation between EDCs with similar chemical structure and their ERE-mediated activities for both ERα and ERβ, but not their known ligand binding affinities. Few EDCs tested in this study weakly induced ERα and ERβ via the “tethered”-mediated mechanism. Using cells stably expressing ERα, we demonstrated that multiple EDCs can differentially induce endogenous ER target genes. Taken together, these data raise a question as to whether multiple assays will be required to assess the potential activity of EDCs. Our results also demonstrate the mechanistic importance of chemical structure similarities and cell type/promoter specificity in the evaluation of potential activities of multiple EDCs.

## Supplemental Material

(606 KB) PDF
