# Supplementary material for: Endocrine-Disrupting Chemicals (EDCs): In Vitro Mechanism of Estrogenic Activation and Differential Effects on ER Target Genes
Source: Environ Health Perspect. 2013 Feb 5;121(4):459–66. doi: 10.1289/ehp.1205951 (PMC3620735; doi:10.1289/ehp.1205951)
Supplement: (606 KB) PDF [file ehp.1205951.s001.pdf]

Supplemental Material

Endocrine-Disrupting Chemicals (EDCs): *In Vitro* Mechanism of Estrogenic Activation and Differential Effects on ER Target Genes

Yin Li, Colin J. Luh, Katherine A. Burns, Yukitomo Arao, Zhongliang Jiang, Christina T. Teng, Raymond R. Tice, and Kenneth S. Korach

Table of Contents

Table S1. EDC information 2

Methods: Construction of the full-length mouse ERβ expression plasmid 3

Figure S1. EDCs act as agonists on a mutant ERβ to activate the classical mechanism (ERE) in HepG2 and HeLa cells. 4

Figure S2. EDCs act as agonists on a mutant ERβ to activate the activate the tethered mechanism (AP-1) in HepG2 and HeLa cells. 5

Reference 6

Table S1. EDC information

EDCs	CAS No.	Supplier
17β-estradiol (E2)	50-28-2	Sigma-Aldrich
ICI 182,780 (ICI)	129453-61-8	Sigma-Aldrich
**Group 1**		
Bisphenol A (BPA)	80-05-7	Sigma-Aldrich
Bisphenol AF (BPAF)	1478-61-1	Sigma-Aldrich
2-2-bis(p-hydroxyphenyl)-1,1,1-trichloroethane (HPTE)	72-43-5	Sigma-Aldrich
4-n-Nonylphenol (4-n-NP)	104-40-5	Alfa Aesar
**Group 2**		
Daidzein (Dai)	486-66-8	Alfa Aesar
Genistein (Gen)	446-72-0	Sigma-Aldrich
Kaempferol (Kaem)	520-18-3	Indofine Chemical Co., Inc
Apigenin (Api)	520-36-5	Sigma-Aldrich
Coumestrol (Coum)	479-13-0	Enzo Life Scinces
**Group 3**		
Endosulfan (Endo)	115-29-7	ChemService, Inc.
Kepone (Kep)	143-50-0	Supelco
1-Bromopropane (1-BP)	106-94-5	Aldrich Chemical Co.

Methods: Construction of the full-length mouse ERβ expression plasmid

The pcDNA3/WT-ERβ plasmid (**pcDNA/WT-ERβ**) was generated as follows, the cDNA fragments of mouse ERβ (mERβ-N-terminal and mERβ-C-terminal) were amplified by PCR using the following primer sets respectively, mERβ-N-ter_5'_BglII; 5'-AGA TCT CTG AGA GCA TCA TGA GCA TCT GTG CCT C-3' and mERβ-N-ter_3'; 5'-CAC TGG TTC TCT TGG CTT TGT TCA GGC AAT GC-3', mERβ-C-ter_5'; 5'-CAA GTG TTA CGA AGT AGG AAT GGT CAA GTG TGG-3' and mERβ-C-ter_3'; 5'-TCT CTG CTT CCT GGC TTG CGG TAG C-3'. The amplified fragments were cloned into pCR2.1 by TA-cloning kit (Invitorogen) and sequenced (NIEHS sequencing core). The KpnI-PstI digested fragment of mERβ-C-terminal was replaced with the KpnI-PstI fragment of pBluescript-mERβ310G-C-terminal plasmid that contains KpnI-XbaI fragment of mERβ cDNA from the pcDNA3/ΔNmERβ310G (**pcDNA/ΔNERβG**) (Mueller et al, 2003) to generate pBluescript-mERβ-C-terminal plasmid. The KpnI-XbaI fragment of pBluescript-mERβ-C-terminal plasmid and the KpnI fragment of pCR2.1-mERβ-N-terminal plasmid were replaced with the KpnI-XbaI fragment of **pcDNA/ΔNERβG** to generate **pcDNA/WT-ERβ** plasmid. The direction of inserted fragment was determined by EcoRI digestion and sequencing.

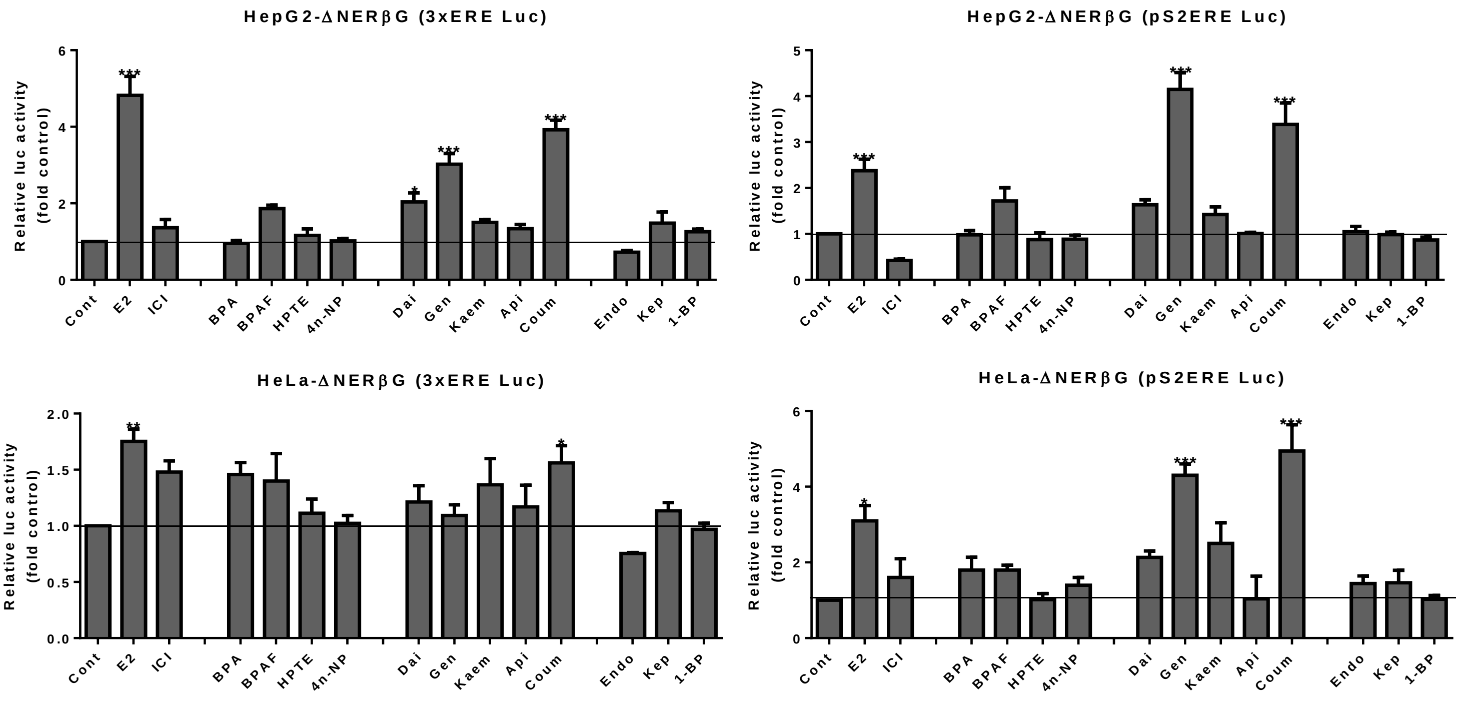

Figure S1. EDCs act as agonists on a mutant ERβ to activate the classical mechanism (ERE) in HepG2 and HeLa cells.

Cells were transfected with ERE-luc (3xERE or pS2 ERE), pRL-TK and pcDNA/ΔNERβG plasmids overnight. After changing to fresh starve medium, cells were treated with the vehicle (control), 10 nM E2, 100 nM ICI or EDCs for 18 hours. ERβ ERE-mediated activation was detected by luciferase reporter assays as described in Material and Methods. Data shown is representative of triplicates as fold increase calculated relative to the vehicle (control) ± SEM, ***, $p < 0.001$, **, $p < 0.01$, *, $p < 0.05$ compared to vehicle (control).

A

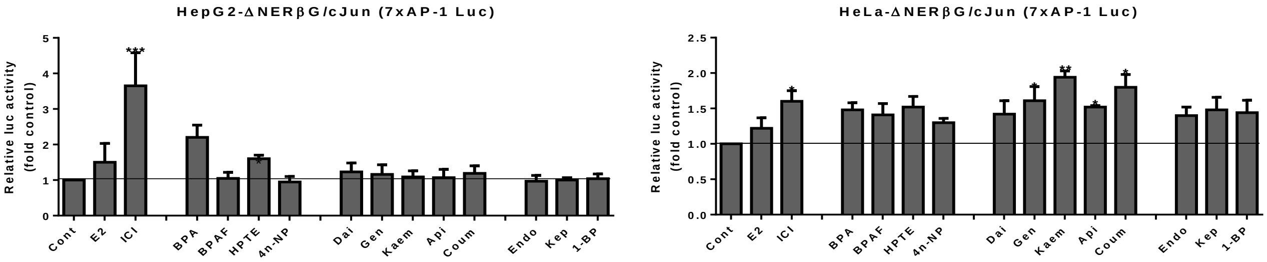

B

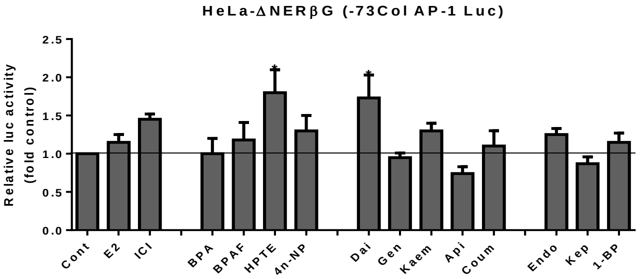

Figure S2. EDCs act as agonists on a mutant ERβ to activate the activate the tethered mechanism (AP-1) in HepG2 and HeLa cells.

(A) Effects on 7xAP-1 Luc reporter activity in HepG2 and HeLa cells. Cells were transfected with 7xAP-1 Luc, pRL-TK, pcDNA/ΔNERβG and pRSV/c-Jun plasmids overnight. After changing to fresh starve medium, cells were treated with the vehicle (control), 10 nM E2, 100 nM ICI or EDCs for 18 hours. ERβ AP-1-mediated activation was detected by luciferase reporter assays as described in Material and Methods. (B) Effects on -73Col AP-1 Luc reporter activity in HeLa cells. Cells were transfected with -73Col AP-1 Luc, pRL-TK, and pcDNA/ΔNERβG plasmids overnight. After changing to fresh starve medium, cells were treated with the vehicle (control), 10 nM E2, 100 nM ICI, or EDCs for 18 hours. ER -73Col AP-1-mediated activation were detected by luciferase reporter assays as described in Material and Methods. Data shown is representative of triplicates as fold increase calculated relative to the vehicle (control) ± SEM, ***, $p < 0.001$, **, $p < 0.01$, *, $p < 0.05$ compared to vehicle (control).

Reference

Mueller SO, Hall JM, Swope DL, Pedersen LC, Korach KS. 2003. Molecular determinants of the stereoselectivity of agonist activity of estrogen receptors (ER) alpha and beta. The Journal of biological chemistry 278(14):12255-12262.
